# Effects of Local Vibration With Different Intermittent Durations on Skin Blood Flow Responses in Diabetic People

**DOI:** 10.3389/fbioe.2019.00310

**Published:** 2019-11-05

**Authors:** Weiyan Ren, Fang Pu, Huiqin Luan, Yijie Duan, Honglun Su, Yubo Fan, Yih-Kuen Jan

**Affiliations:** ^1^Beijing Key Laboratory of Rehabilitation Technical Aids for Old-Age Disability, Key Laboratory of Human Motion Analysis and Rehabilitation Technology of the Ministry of Civil Affairs, National Research Center for Rehabilitation Technical Aids, Beijing, China; ^2^Key Laboratory of Rehabilitation Technical Aids of Ministry of Civil Affair, School of Biological Science and Medical Engineering, Beihang University, Beijing, China; ^3^Advanced Innovation Center for Biomedical Engineering, School of Biological Science and Medical Engineering, Beihang University, Beijing, China; ^4^Rehabilitation Engineering Laboratory, Department of Kinesiology and Community Health, University of Illinois at Urbana-Champaign, Champaign, IL, United States

**Keywords:** diabetes mellitus, diabetic foot ulcers, vibration, skin blood flow, microcirculation

## Abstract

**Objective:** Poor blood flow supply is an important pathological factor that leads to the development and deterioration of diabetic foot ulcers. This study aims to investigate the acute effects of local vibration with varying intermittent durations on the plantar skin blood flow (SBF) response in diabetic and healthy subjects.

**Methods:** Eleven diabetic patients (7 males, 4 females) and 15 healthy adults (6 males, 9 females) participated in this experiment and accepted three tests. Local continuous vibration (LCV) and two levels of local intermittent vibration (LIV1 and LIV2) were randomly applied to the middle metatarsal head of each subject's right foot in each test. The SBF was measured prior to intervention (Baseline), during Vibration and during the Recovery Stage for each test. The mean SBF in each stage, the change percentages and change rates of SBF in Vibration and Recovery stage among three tests were compared and analyzed for both diabetic and healthy subjects.

**Results:** For diabetic subjects, the SBF was significantly increased in both Vibration and Recovery Stage with local intermittent vibrations (LIV1 and LIV2), but not with LCV. However, there was no significant difference in change percentage and change rate of SBF in diabetic subjects across the three tests. For healthy subjects, all vibration interventions significantly increased the SBF in the Vibration Stage and in the first 1.5 min of the Recovery Stage. Also, the change rate of SBF during the Vibration stage in LIV1 test was significantly greater than that in LIV2 test for healthy subjects. Moreover, change percentage of SBF in Vibration stage of LIV1 test and in some periods of Recovery stages of LIV1 and LIV2 tests for diabetic subjects were lower than for healthy subjects; the absolute change rate of SBF in LIV1 test for diabetic subjects was also lower than for healthy subjects.

**Conclusion:** These findings suggest that both LIV1 and LIV2 may effectively improve SBF in the feet of diabetic people, but LCV may not achieve the same level of vasodilatation. The diabetic subjects were also found to have a lower SBF response to applied vibration than the healthy subjects.

## Introduction

Foot ulcers are one of the most serious complications for diabetics (Burns and Jan, 2012). Previous studies have reported that impaired functionality of microcirculation can cause plantar tissue ischemia, and increase the incidence of foot lesions and ulcers (Wiernsperger, 2001; Jan et al., 2013). Thus, it is reasonable to hypothesize that an intervention that can improve the supply of plantar blood flow may reduce the risk of foot ulcers.

Nakagami et al. showed that applying continuous vibration of 47 Hz to the ear of hairless male mice for 15 min could achieve venules vasodilation and promote the healing of ulcers by improving the skin blood flow (SBF) (Nakagami et al., 2007). Yu et al. reported that continuous vibration of low-magnitude high-frequency (not higher than 50 Hz) is beneficial to accelerating the foot wound healing by enhancing blood microcirculation (Yu et al., 2017). Lohman et al. applied short-term local intermittent vibrations of 30 Hz with a cycle of 60-s vibration and 30-s rest to the calves of healthy subjects and found that such intermittent vibrations could improve SBF in the lower limbs (Lohman et al., 2007, 2011). Similarly, Lythgo et al. reported that cycles of 60-s vibration and 60-s rest could increase blood cell velocity in the leg (Lythgo et al., 2009). These positive effects of vibration are related to the release of NO and activation of neural reflex activity induced by pulsating mechanical stimulus of vibration (Sackner et al., 2005; Napoli et al., 2006; Nakagami et al., 2007; Ichioka et al., 2011).

Some studies reported that the interval training may induce a stronger vascular response by generating higher shear forces compared to continuous training, which indicates the intermittent stimulation may be more helpful to inducing physiological responses compared to continuous stimulations (Wisløff et al., 2007; Ribeiro et al., 2010; Mitranun et al., 2014). Nakagami et al. pointed out that continuous vibration over a certain dose may cause adverse effects on nerves and microvasculature (Nakagami et al., 2007). To our knowledge, how skin blood flow responses to continuous and intermittent vibration interventions and what the differences among these microvascular responses in the plantar soft tissue were still unknown. Maloney-Hinds et al. applied vibration interventions of 30 and 50 Hz, respectively, to the forearm of healthy adults for 10 min, and measured SBF for 10 s after every 1-min vibration. Results showed that both 30 and 50 Hz vibration significantly increased the SBF in the forearm, and the peak SBF was reached 5 min after the intervention. Moreover, greater improvement in SBF was achieved when 50 Hz vibration was applied (Maloney-Hinds et al., 2008). A follow-up study by the same group found that local vibrations of 50 Hz for 5 min could significantly increase SBF and nitric oxide (NO) production rate in the forearm of both diabetic and healthy subjects, but the microcirculation parameters in the diabetics were still lower than that for the healthy subjects (Maloney-Hinds et al., 2009). Based on their results described above, the authors deduced that vibration intervention could be used to improve SBF in the feet of diabetic patients. However, the SBF regulation mechanism and response characteristics of microcirculation in the upper limbs are quite different to the lower limbs (Petrofsky et al., 2011), and the vasculopathy of lower limbs in diabetics is typically more serious than that of the upper limbs (Silber et al., 2007). Therefore, the effect of vibration intervention on plantar blood flow in diabetics requires further investigation.

This study aims to explore the acute effects of local vibration interventions on the plantar SBF responses in diabetic and healthy subjects, and further analyze the microcirculation response in diabetics. In accordance with the findings of Maloney-Hinds et al. (2008), 5-min vibrations of 50 Hz with various rest period durations were applied in this study. The effects of different vibration patterns (i.e., with different rest period durations) on microvascular response were compared. The goal was to explore the vibration pattern that could increase plantar blood flow and potentially reduce the risk of developing foot ulcers.

## Methods

### Participants

Fifteen diabetic and 15 healthy adults were enrolled in this study. The inclusion criteria for diabetic subjects were: (i) diagnosed with type 2 diabetes mellitus, (ii) aged 55–75 years, (iii) have no previous history of foot ulcers or amputation, (iv) were never diagnosed with severe complications like peripheral neuropathy, peripheral arterial disease (ankle brachial index (ABI) <0.9), renal disease, retinal disease, liver disease, cancer, or coronary heart disease, and have never had reconstructive vascular surgery. The inclusion criteria for healthy subjects were: (i) no swelling, inflammation or lesions on the feet or legs, (ii) the absence of hypertension, peripheral neuropathy, heart disease, or other vascular diseases. Finally, in total of 11 diabetic people and 15 healthy adults met the criteria and participated in this study. Their demographic information is shown in Table 1. This study was conducted in accordance with clinical protocols approved by the institutional review board of Affiliated Hospital of National Research Center for Rehabilitation Technical Aids. All subjects gave informed written consent prior to participation.

**Table 1:** Demographic and physiological information of the subjects.

**Variables**	**Diabetic subjects**	**Healthy subjects**
Gender (Male/Female)	7/4	6/9
Age (years)	66.45 ± 4.03	22.87 ± 0.64*
BMI (kg/m^3^)	26.05 ± 3.02	20.34 ± 2.62*
SBP	131.18 ± 14.50	120.80 ± 11.51
DBP	69.45 ± 8.95	70.87 ± 8.11
Heart rate	71.36 ± 6.38	76.07 ± 9.57
VPT_WF	7.80 ± 3.57	2.60 ± 1.09*
VPT_MM	6.27 ± 4.00	2.40 ± 1.40*
ABI	1.18 ± 0.13	1.04 ± 0.09*
Fasting glucose (mmol/L)	7.66 ± 1.36	/
HbA1c (%)	7.60 ± 1.35	/

BMI, body mass index; SBP, systolic blood pressure; DBP, diastolic blood pressure; VPT_WF, vibration perception threshold of whole foot; VPT_MM, vibration perception threshold of middle metatarsal; ABI, ankle brachial index. “^*^” means that there was a significant difference in the physiological characteristics between diabetic and healthy subjects.

### Procedures

**Figure 1:**
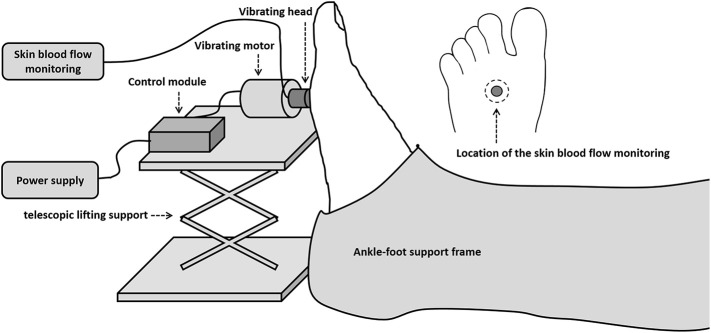
Test setup for measuring skin blood flow (SBF) of the right middle metatarsal.

A custom-designed device was used to apply vibrations to the right middle metatarsal head of each subject. The vibration device consisted of a motor, a vibration head, a control module, a power supply, a lifting platform, and a foot-calf support frame. The design allowed the duration of the stimulus and pause periods to be changed. The vibration head, made with TPE materials by 3D printer, was a hollow cylinder with an outer diameter of 10 mm and an inner diameter of 5 mm. The frequency of vibrations was set as 50 Hz and the amplitude at 2 mm. The frequency of 50 Hz was chosen as in a previous study (Maloney-Hinds et al., 2008). The force generated by the vibrating head was measured by a calibrated pressure sensor and is about 9.45 kPa. A probe from a laser doppler flowmeter (PeriFlux 5001, Perimed, Stockholm, Sweden) was used to measure SBF of the middle metatarsal before, during and after vibration intervention. It was placed within the hole of the cylinder and over the plantar skin during the experiment. The test setup is shown in Figure 1. During the test, the vibrator head was adjusted perpendicular to the area of the middle metatarsal head, and both subjects' feet and vibration device were fixed, ensuring a vertical force stimulus was applied to subjects' feet.

The parameters of three different vibration interventions were set as below: Local Continuous Vibration (LCV) has a continuous vibration for 5 min (the intermittent duration was 0 s). Local Intermittent Vibration 1 (LIV1) consisted of 10 s vibration followed by 5 s pause for a total time of 7.5 min. Local Intermittent Vibration 2 (LIV2) consisted of 10 s vibration followed by 10 s pause for a total time of 10 min. All three interventions have a total vibration time of 5 min.

Before the test, all subjects were asked to rest for 30 min in a room with a temperature of 24 ± 2°C. Before applying any stimulus, SBF of the middle metatarsal was recorded in the supine position for 5 min as the Baseline Stage. Then one of the three vibration interventions was randomly chosen and applied to the middle metatarsal head. The SBF was continuously recorded during vibration (Vibration Stage). Once vibration intervention had ceased, the SBF was continuously recorded for 5 min (Recovery Stage). A 30 min interval was allowed for the subject to rest, and then the process was repeated with the remaining two vibration interventions.

### Data Analysis

The mean values of SBF, change percentages and change rates of SBF in diabetic and healthy subjects were analyzed. The mean values of SBF were calculated for various stages and periods, i.e., Baseline Stage, intermittent (no vibration) durations in Vibration Stage and each 0.5 min in Recovery Stage [marked as Rec (0–0.5 min), Rec (0.5–1 min), Rec (1–1.5 min), etc.], which was described as SBF_baseline_, SBF_vibration_, and SBF_recovery_. The percentage changes and change rates of SBF was calculated for both Vibration and Recovery Stage. The former was computed as Equation (1). The latter was calculated from a linear regression approach as the slope of the regressed line. For Vibration Stage, it was computed from 30 mean values of SBF during successive intermittent (no vibration) durations. For Recovery Stage, it was computed from 30 mean values of SBF for each successive 10 s duration.

(1) $$\begin{array}{r} \frac{{{\text{M}ean\;\text{S}BF}_{\text{s}}-\;\text{M}ean\;\text{S}BF}_{\text{b}a\text{s}eline}}{{\text{M}ean\;\text{S}BF}_{\text{b}aseline}} \end{array}$$

Where “s” represents Vibration or Recovery stage.

Various statistical tests were conducted. The Shapiro-Wilk test was first used to test the normality of the SBF parameters for each stage. For the SBF parameters following a normal distribution, (i) a paired t-test was selected to test the differences in mean SBF parameters, between Baseline Stage and Vibration/Recovery Stage, in order to investigate the immediate and continuous effect of vibration on SBF; (ii) a paired *t*-test was selected to test the differences in change percentage and change rate of SBF during Vibration Stage between LIV1 and LIV2 tests, and an independent *t*-test was selected to test the differences in change percentage and change rate of SBF in three tests, in order to explore the effects of different vibration patterns on SBF responses; (iii) a paired *t*-test was selected to test the differences in change percentage and change rate of SBF between two subject groups, in order to explore the differences of SBF responses to vibration interventions between diabetic and healthy adults. While for the SBF parameters not following a normal distribution, a non-parametric Wilcoxon test or Friedman test was used to test the differences between two or three sets of data, respectively. A statistical significance level of 0.05 was used. All statistical analyses were performed in SPSS (Version 20.0, IBM, Armonk, NY, USA).

## Results

**Figure 2:**
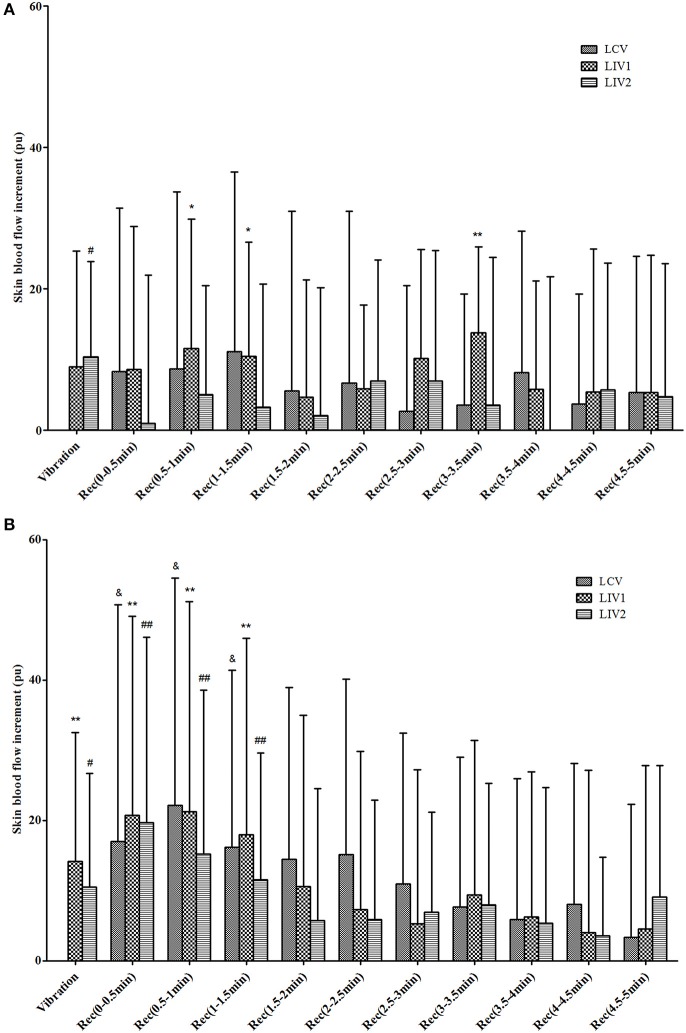
Increments of skin blood flow (SBF) during Vibration and Recovery Stages under three vibration tests in diabetic **(A)** and healthy **(B)** subjects. LCV, Local Continuous Vibration; LIV1, Local Intermittent Vibration 1; LIV2, Local Intermittent Vibration 2. “&” indicates the SBF parameter was significantly greater than basal SBF in LCV test; *P* < 0.05. “*” indicates the SBF parameter was significantly greater than basal SBF in LIV1 test; *means *P* < 0.05, **means *P* < 0.01. “#” indicates the SBF parameter was significantly greater than basal SBF in LIV2 test; # means *P* < 0.05, ## means *P* < 0.01.

The increments of plantar SBF for the different stages under the three vibration interventions in diabetic and healthy subjects are shown in Figure 2. For diabetic subjects, LCV did not increase SBF in the Vibration or Recovery Stage, while LIV1 did significantly increase the SBF during some periods in the Recovery Stage, and LIV2 significantly increased the SBF in the Vibration Stage. The healthy subjects displayed similar SBF responses to the different vibration interventions, i.e., both LIV1 and LIV2 significantly increased the SBF in the Vibration Stage, and all vibration interventions significantly increased the SBF within 1.5 min in the Recovery Stage.

The percentage change in SBF is shown in Table 2. The percentage change of SBF during Vibration stage and Rec (0–0.5 min) for LIV1, and during Rec (0–0.5 min) and Rec (0.5–1 min) for LIV2 for healthy subjects were significantly greater than for diabetic subjects. However, there was no significant difference among the three vibration tests.

**Table 2:** Change percentage of SBF in different stages under three vibration tests in diabetic and healthy subjects.

		**LCV**		**LIV1**		**LIV2**	
		**Diabetic**	**Healthy**	**Diabetic**	**Healthy**	**Diabetic**	**Healthy**
Vibration stage		/	/	26 ± 42%	102 ± 129%^*^	20 ± 31%	43 ± 43%
Recovery stage	0~0.5 min	27 ± 54%	72 ± 112%	23 ± 46%	138 ± 199%^**^	6 ± 47%	76 ± 87%#
	0.5~1 min	27 ± 53%	86 ± 125%	25 ± 39%	138 ± 190%	9 ± 35%	59 ± 69%#
	1~1.5 min	31 ± 62%	60 ± 101%	22 ± 31%	111 ± 170%	8 ± 25%	45 ± 48%
	1.5~2 min	18 ± 37%	57 ± 100%	17 ± 31%	71 ± 145%	5 ± 19%	26 ± 55%
	2~2.5 min	19 ± 40%	66 ± 103%	12 ± 23%	61 ± 135%	9 ± 17%	27 ± 51%
	2.5~3 min	14 ± 40%	56 ± 107%	15 ± 25%	51 ± 134%	10 ± 17%	28 ± 55%
	3~3.5 min	13 ± 35%	41 ± 113%	25 ± 30%	67 ± 131%	4 ± 25%	59 ± 131%
	3.5~4 min	18 ± 34%	41 ± 118%	13 ± 28%	65 ± 142%	2 ± 22%	47 ± 116%
	4~4.5 min	9 ± 23%	44 ± 118%	13 ± 31%	75 ± 186%	7 ± 21%	18 ± 30%
	4.5~5 min	11 ± 24%	34 ± 100%	13 ± 32%	91 ± 191%	5 ± 18%	36 ± 67%

The variations were calculated as the change percentages of SBF in vibration/recovery stage compared to baseline stage. LCV, Local Continuous Vibration; LIV1, Local Intermittent Vibration 1; LIV2, Local Intermittent Vibration 2. SBF, skin blood flow. “^*^” indicates that there was a significant difference in SBF change percentages between diabetic and healthy subjects in LIV1 test.

* means P < 0.05,

** *means P < 0.01. “#” indicates that there was a significant difference in SBF change percentages between diabetic and healthy subjects in LIV2 test. # means P < 0.05*.

The values for change rate of SBF are detailed in Table 3. The change rate of SBF during Vibration Stage in LIV1 in healthy subjects was significantly greater than that in LIV2. Moreover, for healthy subjects undergoing LIV1 stimulation, the results for the Vibration Stage were significantly greater and the results for the Recovery Stage were significantly lower than those for diabetic subjects. This indicated that healthy people have a faster response rate than diabetic people.

**Table 3:** Change rate of SBF in different stages under three vibration tests in diabetic and healthy subjects.

	**LCV**		**LIV1**		**LIV2**	
	**Diabetic**	**Healthy**	**Diabetic**	**Healthy**	**Diabetic**	**Healthy**
Vibration Stage	N/A	N/A	0.010 ± 0.017	0.038 ± 0.023^*^	0.004 ± 0.014	0.021 ± 0.024^$^
Recovery Stage	−0.007 ± 0.016	−0.039 ± 0.127	−0.004 ± 0.012	−0.031 ± 0.060^*^	−0.001 ± 0.012	−0.010 ± 0.020

*LCV, Local Continuous Vibration; LIV1, Local Intermittent Vibration 1; LIV2, Local Intermittent Vibration 2. SBF, skin blood flow. N/A, not applicable. “$” indicates that there was a significant difference in the change rate of SBF for healthy subjects between LIV1 and LIV2 tests*.

$ means P < 0.05. “^*^” indicates that there was a significant difference in the change rate of SBF between diabetic and healthy subjects.

* *means P < 0.05*.

Moreover, the results also demonstrated that the basal SBF in diabetic subjects (83.14 ± 54.60 pu) was significantly greater than in healthy subjects (25.67 ± 20.25 pu) (*P* < 0.001).

## Discussion

This study explored the acute effects of three different vibration interventions on SBF in the feet of diabetic and healthy subjects. This offers a better understanding of how microcirculation responds to vibration, which may lead to more robust decision concerning treatment strategy.

Vessel vasodilation and subsequent increases in the SBF induced by vibration are mainly regulated via two mechanisms: (i) the pulsating mechanical forces act on the endothelial cells to release NO and NO synthase (NOS), which contributes to vessel vasodilation; (ii) Simulation of polymodal receptors on the skin surface by the vibration could cause the release of neuropeptides, and further induce nerve axon reflex-related microvascular vasodilation (Sackner et al., 2005; Napoli et al., 2006; Nakagami et al., 2007). The study of Gailiuniene et al. showed that low frequency 2–10 Hz vibration could not make any significant changes in foot temperature and blood flow (Gailiuniene et al., 2017). While high frequency vibration was reported to be harmful to vascular, musculoskeletal, and nervous system function (Govindaraju et al., 2010; Gailiuniene et al., 2017). According to the previous studies, vibration interventions with frequencies range from 20 to 50 Hz were beneficial to improving blood microcirculation, and 50 Hz vibration may achieve a higher increase of skin blood flow (Lohman et al., 2007, 2011; Nakagami et al., 2007; Maloney-Hinds et al., 2008; Lythgo et al., 2009; Merriman and Jackson, 2009). Thus, we applied 50 Hz vibration as the intervention method, and the results showed that the selected vibration intervention can effectively increase plantar SBF in both diabetic and healthy subjects during and after the application of vibration (as shown in Figure 2), which is in accordance with previous studies (Nakagami et al., 2007; Maloney-Hinds et al., 2009). However, the results of this study indicated that continuous vibration does not significantly increase SBF in diabetics (Figure 2). Nakagami et al. pointed out that although vibration stimuli can induce microvascular vasodilation through nerve axon reflexes, it will cause negative feedback to the nerves once the vibration stimulus exceeds a certain dose, and the microvasculature will no longer dilate (Nakagami et al., 2007). Ma et al. indicated that within a certain range of vibration frequency, as the vibration intensity increased, fewer concentrations of NO and NOS appeared in the tissues of hind legs of rabbits. This phenomenon was speculated to be attributed to the nerve damage caused by vibration (Ma et al., 2007). This could explain, at least partially, the insignificant change of SBF in diabetics with continuous vibration.

Maloney-Hinds et al. investigated the vascular response to vibrations at 50 Hz and amplitude of 5–6 mm applied to the forearms of diabetic and healthy subjects for a period of 5 min. The results showed that SBF increased by 361 and 150% in the Vibration Stage and Recovery Stage in healthy people, respectively, and by 123 and 49% in the Vibration Stage and Recovery Stage in diabetic people, respectively (Maloney-Hinds et al., 2009). In our study, plantar SBF increased by an average of 102 and 87% in the Vibration Stage and Recovery Stage in the LIV1 test in healthy people, and increased by 26 and 18% in the Vibration Stage and Recovery Stage of the LIV1 test in diabetic people (Table 2). A key difference between these two studies is that the former measured SBF in the forearm, whereas the latter measured SBF in the foot. Tissues in the foot are stiffer than that in the forearm, so the microcirculation response may be weaker (Jan et al., 2013). Another potential reason for the difference in results is that the amplitude of vibration used in this study (2 mm) was much lower than in the study of Maloney-Hinds (5–6 mm), which may induce less vessel vasodilation, and thus less increase in SBF. However, the thickness of the plantar soft tissue under middle metatarsal heads is about 8.5 mm, which is much thinner than the forearm tissue (Mickle et al., 2011). Whether the vibration with an amplitude of 5–6 mm would produce a stronger mechanical stimulus and cause detrimental effect to the plantar soft tissue needs further exploration.

Previous studies on intermittent vibration investigated the effects of intermittent whole body vibration on blood flow in legs and blood glucose control, as well as the effect on balance in diabetics. These studies used 30–60 s vibration with 30 s rest or 3 min vibration with 1 min rest (Lee et al., 2013; Del Pozo-Cruz et al., 2014). However, few studies explored the effect of local intermittent vibration on plantar SBF. Maloney-Hinds et al. applied every 1 min vibration with 10 s pause to the subjects' forearm, and found a significant increase of SBF during and after the vibration intervention. Thus, this study set 10 s vibration followed by 0/5/10 s pause as the time regimes of vibration interventions, and explored the effects of varying the pause period in vibration interventions on the SBF responses in diabetic and healthy subjects. Previous studies have shown that the higher the shear forces generated in interval training are, the stronger cellular and molecular responses may be induced (Wisløff et al., 2007; Ribeiro et al., 2010; Mitranun et al., 2014). Although interval exercise training is different from intermittent vibration, we take the principle that continuous stimulations (e.g., continuous exercise and continuous vibration) may induce weaker physiological responses compared to intermittent stimulations (e.g., interval exercise and intermittent vibration). This may be indirectly supported by our results, in which the LIV1 and LIV2 interventions resulted in a greater increase in SBF in diabetic people than LCV intervention. Moreover, LIV1 induced a greater increase in SBF in healthy subjects than LIV2, which suggested that the changes in the length of the intermittent duration result in distinct microvascular responses.

Increased peripheral vascular resistance, inadequate blood supply, and capillary rarefaction often manifest in the microcirculation in diabetic people (Nyberg et al., 2015). Hyperglycemia may lead to decreased deformability, increased aggregation of red blood cells and increased plasma viscosity, which could impair vascular responses (Chao and Cheing, 2009; Kabbani et al., 2013). Moreover, the thickened microvascular intima caused by hyperglycemia would hinder the diffusion of NO from endothelial cells to smooth muscle and reduce the responsiveness of vascular smooth muscle, which may strongly affect the increase of blood flow during vibration (Maloney-Hinds et al., 2009; Ichioka et al., 2011). These may be the reasons why the percentage change and rate of change in SBF for diabetic subjects in this study were relatively weaker than for healthy subjects (Tables 2, 3). In this study, the vibration perception thresholds of 10 regions in diabetic and healthy subjects were measured by a biothesiometer. Although the diabetic subjects recruited in this study did not have peripheral neuropathy, the perception of vibration threshold of the foot in diabetic subjects was significantly higher than that in healthy subjects (whole foot: diabetics: 7.80 ± 3.57 V; Healthy: 2.76 ± 1.12 V, *p* < 0.001; middle metatarsal: diabetics: 6.27 ± 4.00 V; Healthy: 2.67 ± 1.50 V, *p* < 0.01). This indicated the perception of vibration in diabetics is weaker than in healthy people. This may be another reason for the lower SBF response in diabetics than in healthy subjects, as this may affect the neural regulation of blood flow in the microcirculation. The results also show that the basal blood flow in diabetics is significantly greater than that in healthy subjects. This may be due to an impaired sympathetic regulation and arteriovenous shunts in diabetic subjects (Jan et al., 2013), or possibly capillary hyperemia produced by chronic pressure (Newton et al., 2005).

As a non-invasive and safe intervention method, vibration has been applied in the fields of therapy and rehabilitation, and many positive effects were found in improving muscle performance, walking balance and endurance, bone density and so on (Merriman and Jackson, 2009; Rittweger, 2010; Kantele et al., 2015; Fischer et al., 2019). In this study, results showed that the vibration can increase plantar SBF in both diabetic and healthy subjects, indicating that it offers a safe method for improving SBF with few side effects. Therefore, vibration has a wide application range in comparison with hot compress therapy, medicine, and exercise (Maloney-Hinds et al., 2008). In future work, diabetic people with severe clinical symptoms of microcirculatory disorders could be considered.

## Limitations

First, the age and body mass index of the diabetic and healthy groups were unmatched, and may influence the comparison results. However, the vascular sclerosis and insensitive perceptive function usually occurs in elderly and obese people with type II diabetes, and may inherently affect microvascular responses. Moreover, a lower skin blood flow response to applied vibration was observed in diabetic subjects compared to healthy subjects, indicating that impaired vascular function and neural regulation would contribute to a weaker microvascular response to vibration stimulus, which is the point that we want to demonstrate in this study. Thus, we did not consider age and obesity as competing factors in our experiments. More comparisons between elderly people with and without diabetes will explored in the future studies. Second, Lythgo et al. also suggested that both the frequency and amplitude of vibration could affect the blood flow responses (Lythgo et al., 2009), which was not considered in the current study. Moreover, the mid-term and long-term effect could better verify the effectiveness of vibration on promoting peripheral microcirculation, and the contralateral effect helps to indicate whether a local response or systematic response was induced by vibration, which are needed to be further investigated in future studies. Considering the habituation of polymodal receptors to a large vibration stimulus, the parameters of vibration should be investigated to avoid the habituation effect. Moreover, besides epidermal tissue, the perfusion and oxygenation in deep tissue also play an important role in maintaining foot health. The effect of vibration on the function of blood supply both in epidermal and deep tissue needs further researches.

## Conclusion

This study investigated the acute effects of three vibration interventions with different pause durations on plantar SBF response. It was found that intermittent vibration (10 s vibration at a frequency of 50 Hz and amplitude of 2 mm followed by 5 s or 10 s pause) can significantly increase the SBF in both diabetic and healthy subjects, while continuous vibration (50 Hz, 2 mm) can only improve SBF in healthy people. This indicated that the presence of an underlying vascular condition (e.g., diabetes) may have a significant effect on the microcirculation responses to the same vibration intervention. Moreover, the SBF response to vibration in diabetics was weaker than healthy subjects due to the impaired microvascular function.

## Data Availability Statement

All datasets generated for this study are included in the article.

## Ethics Statement

The studies involving human participants were reviewed and approved by Institutional review board of Affiliated Hospital of National Research Center for Rehabilitation Technical Aids. The patients/participants provided their written informed consent to participate in this study.

## Author Contributions

WR, FP, YF, and Y-KJ designed the study and conducted the literature research. WR, HL, YD, and HS carried out experiments, collected data, and performed data analysis. WR, FP, and Y-KJ drafted and revised the manuscript. WR, FP, and YF provided funding. All authors read and approved the final manuscript.

### Conflict of Interest

The authors declare that the research was conducted in the absence of any commercial or financial relationships that could be construed as a potential conflict of interest.
